# *Moramonas marocensis* gen. nov., sp. nov.: a jakobid flagellate isolated from desert soil with a bacteria-like, but bloated mitochondrial genome

**DOI:** 10.1098/rsob.150239

**Published:** 2016-02-17

**Authors:** Jürgen F. H. Strassert, Denis V. Tikhonenkov, Jean-François Pombert, Martin Kolisko, Vera Tai, Alexander P. Mylnikov, Patrick J. Keeling

**Affiliations:** 1 Department of Botany, University of British Columbia, Vancouver, British Columbia, Canada; 2 Institute for Biology of Inland Waters, Russian Academy of Sciences, Borok, Yaroslavl Region, Russia; 3 Department of Biology, Illinois Institute of Technology, Chicago, IL, USA

**Keywords:** *Moramonas*, Moramonadidae, Jakobida, mitochondrion, bloated genome

## Abstract

A new jakobid genus has been isolated from Moroccan desert soil. The cyst-forming protist *Moramonas marocensis* gen. nov., sp. nov. has two anteriorly inserted flagella of which one points to the posterior cell pole accompanying the ventral feeding groove and is equipped with a dorsal vane—a feature typical for the Jakobida. It further shows a flagellar root system consisting of singlet microtubular root, left root (R1), right root (R2) and typical fibres associated with R1 and R2. The affiliation of *M. marocensis* to the Jakobida was confirmed by molecular phylogenetic analyses of the SSU rRNA gene, five nuclear genes and 66 mitochondrial protein-coding genes. The mitochondrial genome has the high number of genes typical for jakobids, and bacterial features, such as the four-subunit RNA polymerase and Shine–Dalgarno sequences upstream of the coding regions of several genes. The *M. marocensis* mitochondrial genome encodes a similar number of genes as other jakobids, but is unique in its very large genome size (greater than 264 kbp), which is three to four times higher than that of any other jakobid species investigated yet. This increase seems to be due to a massive expansion in non-coding DNA, creating a bloated genome like those of plant mitochondria.

## 1. Introduction

The protist group Jakobida (‘jakobids’, within the Excavata) consists of free-living, heterotrophic flagellates that can be found globally distributed in a broad variety of aerobic and anaerobic environments (seawater, freshwater, soil). The small cells with a length of less than 15 µm bear two anteriorly inserted flagella of which one is directed posteriorly (posterior flagellum) and associated with a ventral groove, where bacteria and other food particles get ingested [1–4]. Cysts have been documented for some species (e.g. [4,5]). Unique features of the Jakobida comprise (i) a posterior flagellum with a dorsal vane, (ii) a dorsal fan of microtubules that arise close to the basal body of the anterior flagellum and (iii) a characteristic structure of the non-microtubular fibre, which is dorsally associated with the left microtubular root [4,6]. The monophyletic nature of this group remained unresolved by molecular phylogenetic studies using the SSU rRNA as marker gene alone, but evidence for a common ancestry came recently from analyses of multi-protein datasets (cf. [4,7–11]), suggesting Jakobida as a sister group to a cluster consisting of Euglenozoa, Heterolobosea and Tsukubamonadida (altogether named as ‘Discoba’ [9]). This cluster includes some of the best-known protist species, such as the freshwater flagellates *Euglena* spp. (e.g. [12]) or the vertebrate parasites *Trypanosoma* spp. (e.g. [13]), but to date not even a dozen species of jakobids have been discovered, much less formally described.

Despite the lack of known diversity, jakobids have attracted a great deal of attention due to their mitochondrial genomes, which have been shown to share more characteristics in common with bacteria than any other known mitochondrial genomes. They encode the largest number of genes known (more than 90 assignable genes; more than 60 protein-coding genes), usually including *rpoA–D*, which encode a bacteria-like, four-subunit RNA polymerase that in other eukaryotes has been replaced by a nucleus-encoded, single-subunit, viral enzyme [14–18]. Other typical bacterial features include Shine–Dalgarno translation initiation motifs upstream of the coding regions (not in *Jakoba libera*) as well as similarities in gene order shared with some *α*-proteobacterial operons [14–18]. These characteristics have led to the idea that jakobids may represent one of the earliest-branching eukaryotic lineages [14].

In this study, we combined morphological and molecular phylogenetic data to describe a newly isolated flagellate, *Moramonas marocensis* gen. nov., sp. nov., and determine its phylogenetic position within the Jakobida. To examine the diversity of jakobid mitochondrial genomes, we also sequenced its mitochondrial (mt) DNA, which shares bacterial features found in other jakobids, but is dramatically different in its overall form and size.

## 2. Material and methods

### 2.1. Sampling and culturing

A sample of desert soil was collected by A. A. Abramov (Institute of Physicochemical and Biological Problems in Soil Science, Russia) in March 2007 near Zagora City, Morocco, a small valley separated from the Sahara by a mountain ridge (coordinates: 30°19′50.0″ N, 5°50′17.0″ W). The sample (in a sterile plastic tube) was stored for several years in the dark at 10°C. Pratt medium (0.1 g l^−1^ KNO_3_, 0.01 g l^−1^ MgSO_4_ × 7 H_2_O, 0.1 g l^−1^ K_2_HPO_4_ × 3 H_2_O, 0.001 g l^−1^ FeCl_3_ × 6 H_2_O; pH = 6.5–7.5 [19]) was added to the sample in April 2013 and a single cell of *M. marocensis* was isolated with a micropipette and transferred to a new Petri dish. The culture was then propagated and maintained in Petri dishes filled with Pratt medium and *Pseudomonas fluorescens* bacteria as food. *M. marocensis* is stored at 10°C in the collection of protozoan cultures at the Institute for Biology of Inland Waters, Russia.

### 2.2. Microscopy

Light microscopy was done with a Zeiss Axio Scope A1. For scanning electron microscopy (SEM), flagellates were fixed for 10 min in 2% glutaraldehyde in 0.1 M cacodylate buffer (pH 7.2), washed using the same buffer, and carefully attached to a polycarbonate filter by filtration (diameter: 24 mm; pore size: 1 µm). The attached cells were then dehydrated in a graded series of ethanol and acetone. After critical point drying, the cells were coated with gold–palladium and observed using a JSM-6510LV. For transmission electron microscopy (TEM), cells were fixed on ice for 30–60 min in a cocktail of 0.6% glutaraldehyde and 2% OsO_4_ in 0.1 M cacodylate buffer (pH 7.2), washed using the same buffer, dehydrated in an alcohol and acetone series, and embedded in Spurr's resin [20]. Sections were stained with saturated uranyl acetate and lead citrate [21], and examined with a JEM-1011 electron microscope.

For ultrastructure descriptions, a terminology according to Simpson & Patterson [3] and Yubuki *et al*. [22,23] is used.

### 2.3. Genome sequencing, assembly and annotation

Cells of *M. marocensis* were harvested following peak abundance in culture. Two sampling strategies were used. The first (whole culture) consisted of 7 ml raw culture, while for the second (sorted cells) the same amount of culture was used, but the ratio between target cells and bacteria (prey organisms) was improved by using a BD FACSAria Cell Sorter. The cells were separated from the medium with centrifugal filters (Vivaclear Mini 0.8 µm PES, Sartorius) and subsequently resuspended in the lysis buffer. Genomic DNA was extracted using the MasterPure Complete DNA Purification Kit (Epicentre).

Genomic libraries were prepared with the Nextera DNA Sample Prep Kit (Illumina) and sequenced at McGill University and Génome Québec Innovation Centre (MiSeq, PE, 300 bases). All reads were trimmed with Sickle v. 1.33 [24] using a quality and sequence length threshold of 25 and 150, respectively. After removing transposon sequences, filtered reads of both approaches (whole culture and sorted cells, with each roughly 3 mio paired end reads and 0.85 mio single reads) were combined and tested for the best k-mer value using KmerGenie v. 1.6950 [25]. The combined reads were assembled with Ray v. 2.1.1 [26] and a k-mer of 31 yielding 283 893 contigs. Contigs belonging to *M. marocensis* were detected by BLASTN and TBLASTN searches using Jakobida nucleic acid and protein sequences respectively as query (see below) and the contigs as reference BLAST database [27]. Correct assignments were assured by reciprocal BLAST searches against the entire non-redundant NCBI database. After mapping the reads back to the selected contigs using Bowtie2 v. 2.2.4 [28], the assembly was further visually reviewed and checked for chimeric sequences and duplicated regions with UGENE v. 1.16.2 [29]. Furthermore, in order to detect potential repeated regions, additional self similarity dot plots of the contigs with different word sizes were calculated (not shown). We did not attempt to extend/join the contigs by PCR.

The SSU rRNA gene was found with BLASTN searches against publicly available jakobid SSU rRNA genes. To obtain the nucleus-encoded protein-coding genes for actin, *α*-tubulin, β-tubulin, elongation factor 2 (EF2) and heat shock protein 90 (HSP90), the following steps were conducted for each gene: (i) BLASTP searches using Jakobida protein sequences as query (kindly provided by R. Kamikawa [8]) and translated *M. marocensis* contigs as BLAST database; (ii) aligning of potential candidates against orthologues from Jakobida and other taxa with L-INS-i implemented in MAFFT v. 7.215 [30]; (iii) alignment trimming with trimAl v. 1.4 [31]; and (iv) phylogenetic tree calculation with RAxML v. 7.4.2 [32] using -f a -m PROTGAMMALGF -# 500 parameters. Then, sequences that were unlikely to represent orthologues (showing suspicious phylogenetic positions or long branches) were removed from the untrimmed alignments and, where necessary, protein sequences of real but interrupted orthologues were put together manually. The orthologues were then extracted from the alignments and used for the phylogenetic analyses (see below).

Mitochondrion-encoded genes and open reading frames (ORFs) were detected with MFannot (http://megasun.bch.umontreal.ca/cgi-bin/mfannot/mfannotInterface.pl) and verified with tRNAscan-SE v. 1.21 (for tRNAs; http://lowelab.ucsc.edu/tRNAscan-SE/) and TBLASTN searches of the publicly available mitochondrial protein-coding genes of jakobids (NC_001823, NC_021124–NC_021128) against the identified *M. marocensis* mitochondrion contigs.

Sequences have been submitted to GenBank under accession numbers KT878762 (nucleus-encoded SSU rRNA gene), KU0571693–KU057179 (mitochondrial draft genome).

### 2.4. Phylogenetic analyses

The SSU rRNA gene of *M. marocensis* was aligned together with representatives of other excavates using L-INS-i, and trimmed with trimAl (automated1 mode). One hundred different randomized starting trees were constructed with RAxML using the GTRGAMMA model. For the tree with the highest likelihood, support values were obtained using 500 bootstrap replicates. Bayesian phylogenetic analysis was conducted with MrBayes v. 3.2.5 [33] using nst = 6, rates = invgamma, ngen = 1 000 000, a relative burnin of 25%, and otherwise default settings. Convergence was confirmed with an average standard deviation of 0.00.

The nuclear protein-coding genes of *M. marocensis* were added to five protein sequence datasets (actin, α-tubulin, β-tubulin, EF2 and HSP90) of various protist species [8]. The datasets were extended by orthologues of the jakobid species *Histiona aroides* (kindly provided by R. Kamikawa) and *Andalucia godoyi* (publicly available), aligned and trimmed with L-INS-i and trimAl, respectively, and combined to a single multi-protein alignment. Maximum-likelihood phylogeny was estimated using RAxML with the PROTGAMMALGF model. The best tree was determined based on 100 inferences from different randomized starting trees and support values were inferred from 500 bootstrap replicates. PhyloBayes 3.3f [34] was used for Bayesian-based phylogenetic analysis using -cat -gtr parameters, and otherwise default settings. Convergence was checked with bpcomp -x100 2 parameters (obtained values: maxdiff = 0.25, meandiff = 0.02).

The mitochondrial protein-coding genes of *M. marocensis* were combined with those of further publicly available jakobid species, plus *Tsukubamonas globosa* as outgroup, resulting in 66 protein sequence datasets. Trimmed alignments were combined to a single multi-protein alignment and used for phylogenetic analyses (RAxML and PhyloBayes) as described above. Convergence was confirmed by a largest discrepancy value of 0.00 (maxdiff; PhyloBayes).

## 3. Results

### 3.1. Morphology of *Moramonas marocensis*

**Figure 1.**
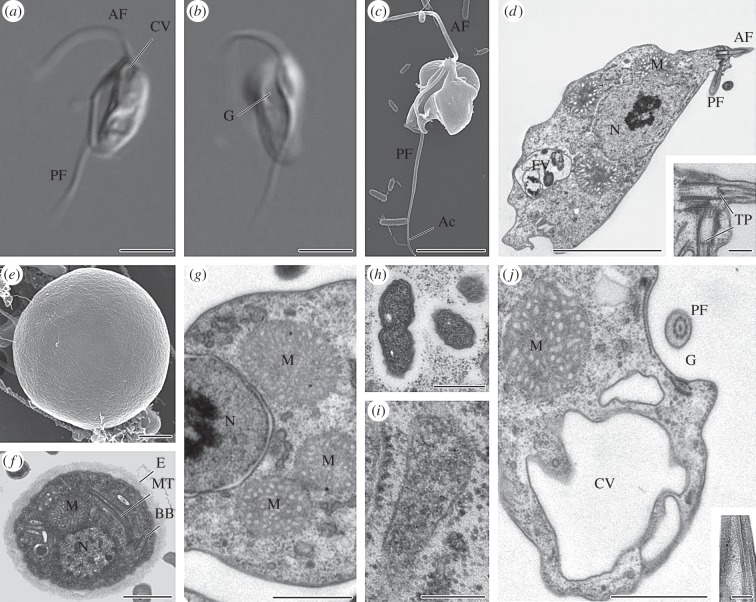
General morphological characteristics of *Moramonas marocensis*. (*a*–*c*) Differential interference contrast (*a*,*b*) and scanning electron microscopy (*c*) showing the flagellate. (*d*) Transmission electron micrograph of a longitudinal section (inset: the anterior cell pole at a higher magnification). (*e*,*f*) Scanning (*e*) and transmission (*f*) electron micrographs showing a cyst. (*g*–*j*) Transmission electron microscopy. (*g*,*j*) Details of an oblique and a transversal section, respectively. (*h*) Intracellular bacteria. (*i*) Microbody. (*j*) Inset: longitudinal section of the posterior flagellum. Ac, acroneme; AF, anterior flagellum; BB, basal body; CV, contractile vacuole; E, envelope; FV, food vacuole; G, ventral groove; M, mitochondrion; MT, microtubules; N, nucleus; PF, posterior flagellum; TP, transversal plate. Scale bars: 4 µm (*a*–*d*), 1 µm (*e–g*, *j*), 0.5 µm (*h*), 0.2 µm (*d* inset, *i*, *j* inset).

**Figure 2.**
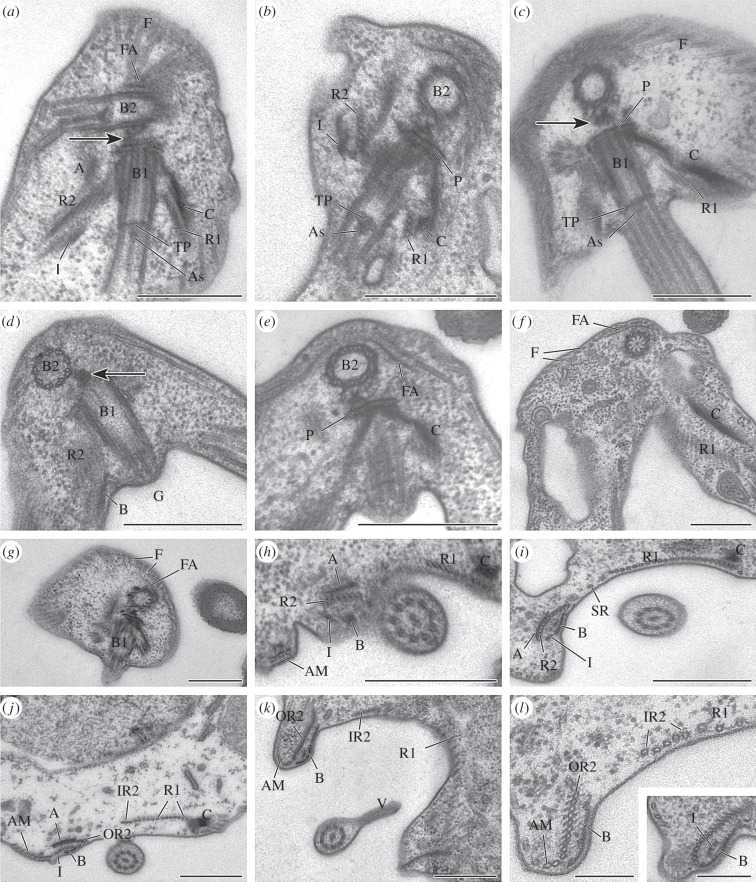
Details of the flagellar apparatus of *Moramonas marocensis*. (*a*–*h*) Transmission electron microscopy of the anterior part of the cell showing the basal bodies of the flagella and their associated root structures. The microtubular right and left roots accompanied by fibres A and C, respectively, and the dorsal fan, are visible. The arrows indicate the fibrils connecting the basal bodies. (*i*–*l*) Transversal sections of the middle part of the cell revealing the divergence of the right root. Further structures, such as fibres A, B, C and I, are conspicuous. A, fibre A; AM, additional microtubules; As, axosome; B, fibre B; B1, basal body of the posterior flagellum; B2, basal body of the anterior flagellum; C, fibre C; F, dorsal fan (dorsal microtubules); FA, fan-associated dense sheet; G, ventral groove; I, fibre I; IR2, internal right root; OR2, outer right root; P, plate close to B1; R1, left root; R2, right root; SR, singlet root; TP, transversal plate; V, vane of the posterior flagellum. Scale bars: 0.5 µm (*a*–*k*), 0.2 µm (*l*, *l* inset).

*Moramonas marocensis* has an elliptical cell shape (figure 1*a–d*) with a length of 7–13 µm (mean 9.6 µm, *n* = 50) and a width of 3–5 µm (mean 4.3 µm, *n* = 50). Spherical cysts with a smooth amorphous envelope (5–6 µm in diameter) can be found 3–4 days after inoculation on the bottom of the Petri dish (figure 1*e*,*f*). The cytoplasm contains elements of the flagellar apparatus (basal bodies and some associated microtubules; figure 1*f*). A lorica is absent. The anterior flagellum (13–14 µm) propels the cell, which usually swims on a straight trajectory rotating around the body axis. The posterior flagellum (16–22 µm) undulates in the ventral groove (e.g. figure 1*b*,*c*,*j*) and has a 2.5 µm long acroneme. It carries a dorsal vane (directed to the ventral groove) with inner striations (figure 1*j* inset, figure 2*k*). Its role in propelling the cell remains unclear. We did not find any connections of the basal bodies (such as rhizoplast) with the nucleus. There is no cytostome.

The single nucleus (2.4–3.8 µm) is located in the centre, slightly anterior and has a central nucleolus. There are three (or less; see Discussion) mitochondria with tubular or—depending on the section orientation—saccular–vesicular-appearing cristae (figure 1*d*,*g*,*j*). A Golgi apparatus has not been found. The cytoplasm may contain small reserve granules and symbiotic bacteria, which are occasionally observed undergoing division (figure 1*h*). The cell possesses a small microbody resembling the paranuclear body in some excavates close to the nucleus (figure 1*i*). At the anterior cell pole, a single contractile vacuole is surrounded by additional smaller vacuoles (figure 1*a*,*j*).

The basal bodies of the anterior and posterior flagellum are approximately orthogonal to each other (figure 2*a*–*d*) and are connected by small fibrils (see arrows in figure 2*a*,*c*,*d*). The axonemes have an ordinary 9 + 2 structure. Both flagella show a transversal plate at the cell surface level just proximal from the axosome (figures 1*d* inset, 2*a*–*c*). The two central microtubules begin close to this structure complex (figure 2*c*). A thin plate is located close to the proximal end of the basal body of the posterior flagellum (e.g. figure 2*b*,*e*). A fan consisting of the dorsal band of microtubules and an associated dense sheet supports the dorsal and lateral sides of the cell (figure 2*a,c*,*e*–*g*).

A right root (R2) consisting initially of five to six microtubules arises close to the basal body of the posterior flagellum (figure 2*a*–*d*). Following its posterior course at the right side of the ventral groove, the number of microtubules increases and the root divides into an internal and an outer rout consisting of 5 and 10 microtubules, respectively (figure 2*h*–*l*). It is accompanied by fibre A on one side and fibres B and I on the other (ventral) side (figure 2*h*–*l*). The right side of the ventral groove shows an expansion that is reinforced by the outer part of the right root. Together with fibre B, this root forms a loop in the posterior part of the right rim of the ventral groove (figure 2*k*,*l*,*l* inset). Additional microtubules close to the outer right root are visible and increase in number more posteriorly (figure 2*j*–*l*).

A left root (R1) consisting initially of four densely packed microtubules arises close to the basal body of the posterior flagellum and is accompanied by fibre C (figure 2*a*–*c*,*e*,*f*,*h*–*j*). The number of microtubules of the left root increases (greater than 15) more posteriorly (figure 2*a*–*c*,*f*,*h*–*k*). The groove floor is supported by microtubules of the left root (figure 2*k*). A singlet root (one microtubule) occupies the space between the right and left root (figure 2*i*).

### 3.2. Phylogenetic analyses

**Figure 3.**
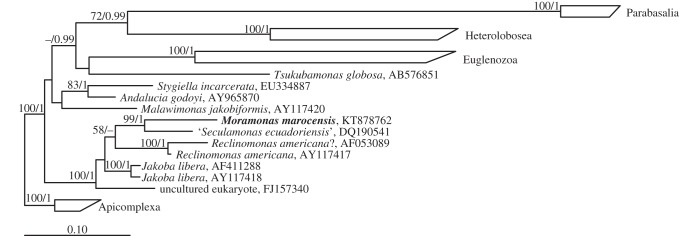
Phylogenetic position of *Moramonas marocensis* (in bold) within the Excavata. Tree topology is based on maximum-likelihood analysis of the SSU rRNA gene sequence dataset. RAxML bootstraps and MrBayes posterior probabilities indicate node supports for values greater than 50 and greater than 0.9, respectively. Missing information or dashes indicate lower values considered as insignificant supports. *Reclinomonas americana* (AF053089) may represent a distinct not formally described species within the *Reclinomonas* genus (see Discussion).

**Figure 4.**
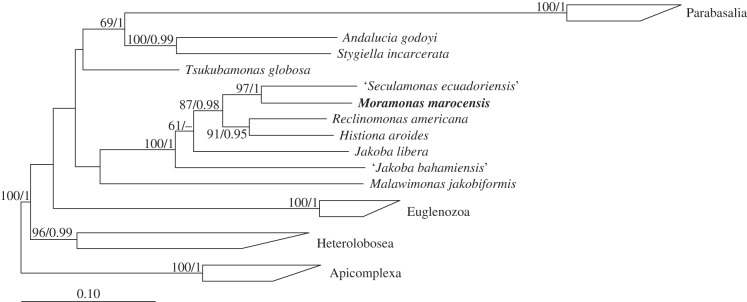
Phylogenetic position of *Moramonas marocensis* (in bold) within the Excavata inferred from a multi-protein dataset. The tree topology is based on maximum-likelihood analysis of the multi-protein (actin, α-tubulin, β-tubulin, EF2 and HSP90) dataset. Node support is indicated by RAxML bootstraps and PhyloBayes posterior probabilities for values greater than 50 and greater than 0.9, respectively. Missing information or dashes indicate lower values (i.e. insignificant supports).

The phylogenetic analysis of the SSU rRNA gene obtained from *M. marocensis* reveals that this flagellate belongs to the group Jakobida within the supergroup Excavata (figure 3). The affiliation to this group was also confirmed by the ML analysis of a dataset including sequences of all major eukaryotic lineages (not shown). The next relative of *M. marocensis* is represented by ‘*Seculamonas ecuadoriensis*’, a flagellate not formally described yet (see Discussion). Although only distantly related (greater than 15% sequence divergence), the branching point between these two jakobids is highly supported. As shown in previous studies [4,11], however, monophyly of the jakobids was not recovered in SSU rRNA phylogenies, with *Andalucia* and *Stygiella* branching separately from the other jakobids. To verify the relationship found in the rRNA tree, the phylogenetic position of *M. marocensis* (figure 4) was also analysed using a multi-protein alignment (actin, *α*-tubulin, *β*-tubulin, EF2 and HSP90; 2645 amino acid positions, 21.67% gaps). This tree confirms that ‘*S. ecuadoriensis*’ is the closest relative of *M. marocensis*, and that both are related to *Reclinomonas americana* (and *H. aroides*). As with the SSU rRNA gene tree, these taxa form a monophyletic cluster that is a sister group to *J. libera* (figures 3 and 4). ‘*Jakoba bahamiensis*’, a flagellate currently lacking formal description and whose SSU rRNA gene was not available (and therefore is not shown in figure 3), does not cluster with *J. libera* but forms a distinct lineage within the jakobids (figure 4). Again, there is no significant support for the phylogenetic affiliation of *Andalucia* and *Stygiella* to any other excavate group, even when the long-branching parabasalids and the ‘problematic’ marker gene *α*-tubulin [10] are omitted from the analysis (not shown).

### 3.3. The mitochondrial genome of *Moramonas marocensis*

**Figure 5.**
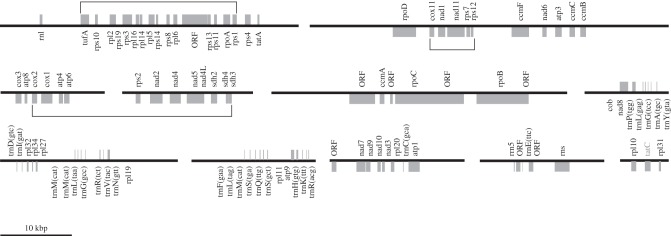
Gene distribution across the eleven contigs of the mtDNA of *Moramonas marocensis*. Boxes above and below the black lines (contigs) indicate genes that are transcribed from left to right and from right to left, respectively. The squared brackets indicate synteny blocks typical for Jakobida. The gene order of the largest block (top left) is comparable with the contiguous S10, Spc and Alpha operons of most *α*-proteobacteria, but shows some gene deletions occurring also in other Jakobida (cf. [18]). *tatC*, a putative pseudogene, is labelled in grey.

We found eleven contigs belonging to the mitochondrial genome of *M. marocensis* with a total size of 264 512 bp (figure 5). The A + T content of these contigs is 72.5%. It contains 90 genes with an almost complete set (97.67%) of 86 core genes present in all other jakobid mitochondrial genomes [18], including the bacteria-like, four-subunit RNA polymerase. There are no identifiable genes that are unique to *M. marocensis*, although it does encode several ORFs that may represent expressed genes (see below). The genome uses the bacterial, archaeal and plant plastidal genetic code (genetic code 11). Almost all genes for the minimal set of tRNAs were found, the exception being *trnT* and *trnW*. These may be encoded in fragments missing from the draft genome, or could be imported from the cytosol [35,36]. A detailed record of mitochondrial genes found in *M. marocensis* is shown in table 1.

**Table 1.** Genes present in the mtDNA (draft genome) of *Moramonas marocensis*.

proteins and structural rRNAs	genes^a^
electron transport and ATP synthesis	
NADH dehydrogenase (complex I) subunits	*nad1*,*2*,*3*,*4*,*4 L*,*5*,*6*,*7*,*8*,*9*,*10*,*11*
succinate dehydrogenase (complex II) subunits	*sdh2*,*3*,*4*
cytochrome *bc* 1 complex (complex III) subunits	*cob*
cytochrome *c* oxidase (complex IV) subunits	*cox1*,*2*,*3*
ATP synthase (complex V) subunits	*atp1*,*3*,*4*,*6*,*8*,*9*
translation	
SSU ribosomal proteins	*rps1*,*2*,*3*,*4*,*7*,*8*,*10*,*11*,*12*,*13*,*14*,*19*
LSU ribosomal proteins	*rpl2*,*5*,*6*,*10*,*11*,*14*,*16*,*19*,*20*,*27*,*31*,*32*,*34*
elongation factor	*tufA*
ribosomal RNAs (LSU rRNA, SSU rRNA, 5S rRNA)	*rnl*, *rns*, *rrn5*
transfer RNAs^b^	*trnA*,*C*,*D*,*E*,*F*,*G*,*H*,*I*,*K*,*L*,*M*,*N*,*P*,*Q*,*R*,*S*,*V*,*Y*
transcription	
core RNA polymerase	*rpoA*,*B*,*C*
sigma-like factor	*rpoD*
protein import	
ABC transporters	*ccmA*,*B*,*C*
SecY-independent transporters	*tatA*,*C*^c^
protein maturation	
cytochrome *c* oxidase assembly	*cox11*
haem C maturation	*ccmF*

^a^*trnW* and *rnpB* (ecoding RNase P) present in the mtDNA of all other jakobids [18] are missing.

^b^There are several variants for the genes *trnG*, *trnL*, *trnR* and *trnS*.

^c^Putative pseudogene (insertion of a single ‘T’ in the nucleotide sequence causes frame shift).

**Figure 6.**
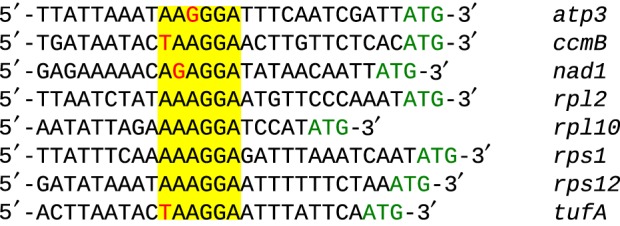
Shine–Dalgarno translation initiation motifs. The putative motifs upstream of some representative genes are highlighted in yellow. The motifs are complementary to a sequence at the 3′ end of the mtSSU rRNA (3′-UUUCCU-5′). A single mismatch is indicated by a red base. The beginning of each gene is indicated by the green initiation codon ATG.

Based on the gene density of these contigs, the predicted genome size of *M. marocensis*'s mitochondrion is over 270 000 bp, which is 3–4 times higher than that of all other known jakobids, resulting in a significantly lower proportion of coding DNA (25% without ORFs in contrast to 80–93% in other jakobids [18]). Eight ORFs were found in addition to the genes identified by sequence similarity to orthologues. Shine–Dalgarno translation initiation motifs (5′-AAAGGA-3′ or highly similar sequences; figure 6) matching the pyrimidine-rich 3′ terminus of the mtSSU rRNA (5′-CUCCUUU_OH_), however, were not only found upstream of several protein-coding genes (greater than 20 with a 100% match), but also of half of the ORFs (one with a 100% match), suggesting that some of them may represent further functional genes.

Although the mitochondrial draft genome consists of 11 linear contigs, synteny blocks of more than 4 genes present also in other jakobid chromosomes were detectable (figure 5). The longest synteny block consists of 15 genes and shows a comparable gene order with the contiguous S10, Spc and Alpha operons of most *α*-proteobacteria (excluding *tufA*, which belongs to the Str operon). In addition to the deletions found in other jakobid mitochondria (e.g. *rpl3*, *rpl4*, *rpl12*, *rps5*), *M. marocensis* shows also the same insertion of a gene cluster encoding *cox11*, *nad1* and *nad11* (but not *cox3*, which is located on a different contig; cf. figure 5 with figures 2 and S2 in Burger *et al*. [18]). Another large synteny block common to jakobids involves two contigs, and comprises genes encoding succinate and NADH dehydrogenase subunits as well as ribosomal protein S2 and ATP synthase F_O_ and cytochrome *c* oxidase subunits (figure 5).

**Figure 7.**
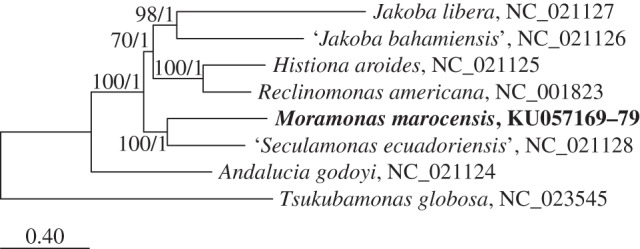
Phylogeny of jakobids inferred from a 66-protein alignment. The tree topology is based on maximum-likelihood analysis of a multi-protein dataset and node support is given for RAxML bootstraps values greater than 50 and PhyloBayes posterior probabilities greater than 0.9. The non-jakobid *Tsukubamonas globosa* represents the ‘outgroup’.

A further phylogenetic analysis was conducted for the mtDNA-encoded proteins using 66 concatenated sequences (15 682 amino acid positions, 8.35% gaps) from jakobids and *T. globosa* as outgroup. The topology of the resulting tree confirms once more that ‘*S. ecuadoriensis*’ is a distant sister to *M. marocensis* (figure 7). Moreover, the distant relationship of the jakobid genus *Andalucia* to jakobids belonging to the Histionina (see Discussion) is also reflected in the long branch separating them. In contrast to the phylogenetic trees obtained from the nucleus-encoded genes, however, *M. marocensis* and ‘*S. ecuadoriensis*’ do not cluster with *R. americana* and *H. aroides* (figure 4), but form a sister group to a cluster consisting of the latter two species plus *Jakoba* spp. (figure 7).

## 4. Discussion

We isolated *M. marocensis* gen. nov., sp. nov., a new jakobid lineage, from desert soil. By combining ultrastructural investigations with molecular phylogenetic analyses of the SSU rRNA gene, five nuclear and 66 mitochondrial protein-coding genes, we here demonstrated the flagellate's phylogenetic affiliation to the Jakobida and reported on its unique characteristics.

### 4.1. Morphological discrimination of *Moramonas marocensis*

*Moramonas marocensis* shows typical morphological features of jakobids: a dorsal fan that arises right next to the basal body of the anterior flagellum and a posterior flagellum with a single dorsal vane with inner striations [3,4,6,37]. Another distinguishing feature for this group is a multilayered substructure of the C fibre [3,4,6] that was less obvious in the sections we investigated. A combination of several morphological characteristics allows the separation from the other Jakobida genera: *M. marocensis* differs from marine jakobids by the presence of a contractile vacuole. It can be distinguished from *R. americana* and *H. aroides* by the absence of a lorica. By the presence of a microbody (paranuclear body), *M. marocensis* can further be distinguished from all other jakobids with the exception of *A. godoyi* [4]. The number of mitochondria might be another distinguishing feature since no other jakobid investigated yet harbours three or more mitochondria. Although it is missing serial cross sections, our investigation of numerous sections of different orientations suggests that there are three spherical mitochondria lacking connecting branches as shown for the mitochondrion in *R. americana* [1].

### 4.2. Phylogeny of Jakobida

The phylogenetic analyses conducted in this study confirmed that *M. marocensis* belongs to the Jakobida. The next (although distant) relative is ‘*S. ecuadoriensis*’, an isolate that has not been formally described, but whose SSU rRNA sequence was published by Lara *et al*. [4]. While ‘*S. ecuadoriensis*’ is the nearest known sister to *M. marocensis,* they are in fact rather distantly related, and together with the missing morphological information about ‘*S. ecuadoriensis*’ this led us to establish a new jakobid species and genus (see below). In the case that morphological investigation of ‘*S. ecuadoriensis*’ reveals a high degree of morphological similarity with *M. marocensis*, their unification within the same genus will have to be considered. Since *Moramonas* cannot be assigned to any of the existing jakobid families, we also propose a new family called Moramonadidae (fam. nov.). This family includes *Moramonas* and ‘*S. ecuadoriensis*’.

The monophyly of the Jakobida could not be demonstrated. In both the maximum-likelihood analysis of the SSU rRNA genes and the maximum-likelihood analysis of five protein-coding genes (figures 3 and 4, respectively), *Andalucia* and *Stygiella* did not cluster with the remaining jakobids. An alternative branching of *A. godoyi* and *Stygiella incarcerata* (formerly known as *Andalucia incarcerata*, and before that as *Jakoba incarcerata* [4]) has also been documented in other studies based on SSU rRNA gene sequence analyses [4,38]. However, neither in these studies nor in our SSU rRNA gene-based analysis did the paraphyletic position of these two species (that could also be discovered when the long-branching parabasalids were excluded from the analysis) show any support. Also, they show all the autapomorphies of Jakobida [3,4], and *S. incarcerata* has recently been demonstrated to cluster with jakobids based on an extensive phylogenetic analysis of a 157-protein dataset [8]. In an even more recent study (the genes sequences were still not publicly available during the revision process of our paper), Pánek *et al*. [11] established a new jakobid family (Stygiellidae), which includes *S. incarcerata* and forms a sister group to the Andaluciidae (both together: Andalucina). Once more, however, monophyly of Andalucina and all other jakobids (known as Histionina) could not be revealed by a SSU rRNA gene sequence analysis but by a phylogenetic multi-protein sequence anlysis [11]. Taking into account these findings, we tend to believe that *Andalucia* and *Stygiella* belong to the Jakobida and that their position in our analyses is caused by insufficient data. The paraphyly (although without support) of the two *Jakoba* species presented in our five protein-coding gene tree was also recovered in the analysis of 157 proteins [8]. Morphological investigations of ‘*J. bahamiensis*’ (a flagellate not formally described yet) will be needed to reassess its genus affiliation.

In the phylogenetic analysis of both the SSU rRNA gene sequence and the protein-coding genes, *M. marocensis* and ‘*S. ecuadoriensis*’ form a sister clade to *R. americana*, which is in agreement with the tree shown by Kamikawa *et al*. [8]. There is no reference reporting the phylogenetic position of *H. aroides* based on a SSU rRNA gene sequence analysis, and its SSU rRNA gene sequence is not publicly available. Its well-supported position in our protein-based phylogenetic analysis, however, suggests *H. aroides* to be the sister to *R. americana* (cf. [11]). The branch lengths between the two *R. americana* species raises the question whether these two jakobids in fact represent only a single species. Indeed, in the original description of one of the two genes, which in GenBank both refer to as *R. americana* (AY117417 [39]; AF053089 [38]), it was referred to as *Reclinomonas* sp., and was described as differing from *R. americana* by showing a ‘high density and greater length of lorica spines’ [38].

The phylogeny of Jakobida inferred from the analysis of 66 mitochondrial protein-coding genes once more placed *M. marocensis* next to ‘*S. ecuadoriensis*’. The topology of the remaining jakobids is in agreement with a phylogenetic tree based on mitochondrion-encoded proteins of jakobids and other taxa shown by Burger *et al*. [18]. The major difference between nuclear gene trees and the mitochondrial gene tree is that *M. marocensis* and ‘*S. ecuadoriensis*’ do not cluster with *Reclinomonas* (and *Histiona*; figure 4), but form a sister group to *Jakoba, Reclinomonas* and *Histiona* collectively.

### 4.3. The bacteria-like, but bloated mitochondrion of *Moramonas marocensis*

During the evolutionary development of mitochondria from a bacterial endosymbiont, its genome underwent an extreme loss of genes due to simple deletions, migration of genes to the nucleus or substitution of genes by nuclear-encoded equivalents. Beyond this reduction, the mitochondrial genome has also been heavily modified in a number of ways in various lineages (see Smith & Keeling [40] for review). As shown for other jakobids [18], the mitochondrial genome of *M. marocensis* possesses several bacteria-like features: a high number of genes, specific bacteria-like gene orders, Shine–Dalgarno sequences and a bacteria-type four-subunit RNA polymerase that in other eukaryotes is substituted by a nucleus-encoded, single-subunit viral enzyme. Moreover, highest BLASTN scores for the SSU rRNA gene sequence can be obtained from bacteria-like mitochondria of jakobids [18] and *α*-proteobacteria with sequence similarities of at least 86% and up to 85%, respectively. Thus, as shown for other jakobids [14,18], the mitochondrial DNA resembles more closely the genomes of *α*-proteobacteria, the closest relatives of the ancestral ‘proto-mitochondria’ endosymbionts (e.g. [41–43]), than the mitochondrial genomes of any other eukaryotic lineage.

There are, however, a few aspects of the *M. marocensis* mitochondrial genome that are not like all other jakobids. First, it appears to be missing the RNase P RNA gene (*rnpB*), which functions in the endonucleolytic cleavage of precursor sequences from the 5′ ends of pre-tRNAs. Evidence has shown that the pre-tRNA cleavage is not active in all Jakobida [18]. However, this absence of a gene may really represent a difference, or *rnpB* may simply be in the small unsequenced portion of the genome.

However, the most striking feature of the *M. marocensis* mitochondrial genome, which is its size, is not in question due to missing data. It is three to four times bigger than any other jakobid mitochondrial genome, despite encoding a comparable set of genes [18]. The size difference is also not caused by introns, but instead is due to a great deal of additional non-coding DNA between the genes, a phenomenon best known from mitochondria of higher plants. Why plant mitochondria genomes have such a bloated, gene-poor form is still debated; some analyses suggest invasion of extra-organellar DNA [44], or cite correlation with mutation rates [40]. The unusually large genome size of the *M. marocensis* mitochondrion appears to be a relatively recent change from a more canonical, gene-dense form since *M. marocensis* is phylogenetically nested within a clade that shares this overall form. Whether this bloating is due to similar causes to that of the land plant mitochondria remains unclear at this point, but there is no evidence for excessive repetitive sequences or repeat-mediated recombination, as known for some plant mitochondrial genomes [45,46], and no evidence for an invasion of detectably extra-organellar DNA. Whether the mitochondrion of *M. marocensis* consists of several or only one circular (most jakobids [18]) or linear (*Jakboa libera* [18]) chromosome is also not clear. The gene set characterized so far suggests that almost the entire mitochondrial genome has been sequenced. Taking into account the high number of large contigs (11), it certainly is possible the genome is made up of several circular and/or linear mtDNA molecules (chromosomes), as has been shown for other protists [47,48]. This too would represent a relatively recent change and its causes are not immediately obvious (see reviews by Burger *et al*. [49] and Smith & Keeling [40]).

### 4.4. Taxonomic summary

In this study, we establish a new jakobid genus and species based on morphological and molecular phylogenetic analyses. We furthermore propose a new family including *Moramonas* and ‘*Seculamonas*’.

Excavata: Discoba: Jakobida: Histionina.

**Moramonadidae fam. nov.:** Aloricate Histionina (groove floor supported by R1 [50]) flagellates with tubular mitochondrial cristae. Type genus: *Moramonas* gen. nov.

***Moramonas* gen. nov.:** Jakobid with two anteriorly inserted flagella without mastigonemes. The cell possesses a microbody close to the nucleus. Extrusomes are absent. The spherical cyst lacks an aperture and plug. Type species: *Moramonas marocensis* sp. nov.

***Moramonas marocensis* sp. nov.:** Cell with a length of 7–13 µm and a width of 3–5 µm. The anterior flagellum measures 13–14 µm in length. The cell swims in a straight line rotating around the body axis. The posterior flagellum (16–22 µm long) has a 2.5 µm long acroneme. The nucleus measures 2.4–3.8 µm in diameter. A contractile vacuole surrounded by additional smaller vacuoles is located at the anterior cell pole. The spherical cyst (5–6 µm in diameter) shows a smooth amorphous envelope.

**Etymology:** Mo.ra.mo'nas. L. f. n. mora—delay, pause; referring to the flagellates' capability to outlast adverse environmental conditions as a cyst. Anc. Gr. f. n. monás (*μ*ο*ν*ά*ς*)—unit. N.L. f. n. Moramonas—a cyst-forming unicellular organism. The species epithet marocensis (ma.roc.en'sis) refers to the place of its discovery, Morocco (L. n. Marocum).

**Type locality:** Organisms were collected from desert soil sample taken near Zagora City, Morocco (30°19′50.0″ N, 5°50′17.0″ W).

**Type material:** One TEM block containing active cells and cysts is deposited at the Beaty Biodiversity Museum (Vancouver, British Columbia, Canada; reference number: MI-PR205), and represents the name-bearing type (a hapantotype). A cell culture is deposited at the Institute for Biology of Inland Waters (Borok, Yaroslavl Region, Russia; reference number: OF-50).

**Gene sequence:** SSU rRNA gene accession number KT878762.

**Zoobank registration:** urn:lsid:zoobank.org:act:D5FDAEBB-C755-4891-88C5-73CDF5943E06, urn:lsid:zoobank.org:act:2626B2E0-82C5-4F88-BCAA-98BF88EE2841, urn:lsid:zoobank.org:act:C435E915-3B0A-4368-9145-2D9D6B564A41.
